# Prognostic factors and outcomes of primary transitional cell carcinoma of the ureter: a population-based study

**DOI:** 10.18632/oncotarget.19623

**Published:** 2017-07-27

**Authors:** Tao Ding, Zhuojun Zheng, Renfang Xu, Cuixing Zhou

**Affiliations:** ^1^ Department of Urology, Third Affiliated Hospital of Soochow University, Changzhou, Jiangsu, China; ^2^ Department of Hematology, Third Affiliated Hospital of Soochow University, Changzhou, Jiangsu, China

**Keywords:** primary transitional cell carcinoma of the ureter, upper tract urothelial carcinomas, SEER, surgery, radiation

## Abstract

**Objective:**

This study investigated the prognostic factors and outcomes of a large observational cohort of patients with primary transitional cell carcinoma of the ureter, which was obtained from the Surveillance, Epidemiology, and End Results database.

**Methods:**

We used the Surveillance, Epidemiology, and End Results program to identify 1910 patients who had available clinical and follow-up information and were diagnosed for primary transitional cell carcinoma of the ureter between 2004 and 2013. Descriptive statistics were used to explore the epidemiology, treatment practices, and tumor characteristics of the patients. Univariate and multivariable Cox regression models were used to analyze the patient data.

**Results:**

The median overall survival (OS) was 46 months, and the 5-year OS rate was 41.8%. The median CSS was 78 months, and the 5-year CSS rate was 54.3%. Multivariate analysis identified tumor grade, tumor size, AJCC stage, M stage, cancer-directed surgical procedure and radiation as independent factors of primary transitional cell carcinoma of the ureter. For early stage patients, the surgical procedure was associated with fairly longer survival and additional radiation may cause more harm than benefit. Meanwhile, for advanced stage patients, the impact of surgery on OS and CSS greatly decreased. Radiation exerted a very limited impact on clinical outcomes. Patients with bad tumor differentiation or a large tumor size were more likely to have advanced stage disease.

**Conclusion:**

Durable cancer control can be expected in patients treated with surgery for early stage UTUC. The presence of advanced stage disease exerts a profound detrimental effect on the survival of patients.

## INTRODUCTION

Primary transitional cell carcinoma of the ureter is one of the main types of primary upper tract urothelial carcinomas (UTUCs), which are rare and heterogeneous diseases that account for approximately 5% of all urothelial tumors [1, 2]. Since 2004, the estimated annual incidence of transitional cell carcinoma of the ureter is only 1.8 cases per 100,000 person-years in the United States, according to the rate session in the SEER statistic. More men (2.7/100,000) than women (1.1/100.000) are likely to be affected. Primary transitional cell carcinoma of the ureter is an aggressive disease with a high progression rate, as over half of cases are invasive and approximately one-quarter of them have a regional metastasis at diagnosis compared to 15% and 20% for all bladder tumors [3–5]. Most patients with this disease have a single ureter affected, and the disease affects both ureters in only 2–4% of patients.

Until recently, there have been few high-quality recommendations to guide physicians in the management of primary transitional cell carcinoma of the ureter. Its low incidence results in small study cohorts, which lead to a lack of good evidence-based data. Because of its biologic heterogeneity, prognosis and different treatment options, primary transitional cell carcinoma of the ureter makes treatment decisions difficult. Recommendations for the evaluation and treatment of primary transitional cell carcinoma of the ureter are mainly based on extrapolations of conclusions from high-evidence-level trials performed in patients with urothelial carcinoma of the lower urinary tract. The cancer-directed surgical procedure is considered to be the gold standard of treatment [1, 2]. Adjuvant radiation and chemotherapy should also be considered in patients with high-risk disease, while patients with low-risk disease may benefit from a more conservative approach, according to the National Comprehensive Cancer Network guidelines [6]. However, biological and molecular differences do exist between the upper and lower urinary tracts. There has not been a thorough study of primary transitional cell carcinoma of the ureter with an exploration of prognostic factors and treatment outcomes in large populations. We believe that a better understanding of the risk factors and treatment options for primary transitional cell carcinoma of the ureter would help to more reasonably guide physicians and establish a more holistic approach to improve patient outcomes. Therefore, in this study, we aimed to explore the prognostic factors and impact of treatments on the clinical outcomes of patients using the National Cancer Institute’s Surveillance, Epidemiology, and End Results (SEER) database.

## RESULTS

### Baseline characteristics

A total of 1910 eligible patients were identified according to the inclusion criteria, including 1114 male and 796 female patients. Ages ranging from 30 years to 101 years were analyzed, with most patients >65 years of age. The median age at diagnosis was 74 years. Most of the included patients were Caucasus. Most of the tumors were undifferentiated or anaplastic (45.4%) and less than 3 centimeters in size (40.4%). There was no significant difference in tumor laterality. The most common T stages were T1 (35.2%) and T3 (29.2%), and 89.0% of the cases were node-negative. Distant metastases were absented in most patients (93.4%). Thus, patients had a larger proportion stratified as American Joint Committee on Cancer (AJCC) stage I (35.2%). The majority of patients (91.9%) underwent a cancer-directed surgical procedure. Only 146 (7.6%) patients underwent radiation. Most of the patients (85.6%) received a surgical procedure only. The demographics and clinicopathological and treatment characteristics are summarized in Table 1.

**Table 1:** Summary of characteristics for the patient population. SEER 2004-2013 (n=1910)^a^

Characteristic	All patients no. (%) (n=1910)
Age	
<65	437(22.9)
65-74	604(31.6)
75-79	358(18.7)
≥80	511(26.8)
Sex	
Male	1114(58.3)
Female	796(41.7)
Race	
Black	67(3.5)
White	1652(86.5)
Other (American Indian/AK Native, Asian/Pacific Islander)	191(10)
Grade	
Well differentiated; Grade I	111(5.8)
Moderately differentiated; Grade II	326(17.1)
Poorly differentiated; Grade III	605(31.7)
Undifferentiated; anaplastic; Grade IV	868(45.4)
Laterality	
Right - origin of primary	943(49.4)
Left - origin of primary	967(50.6)
AJCC stage	
I	635(33.2)
II	472(24.7)
III	450(23.6)
IV	353(18.5)
T stage	
T1	672(35.2)
T2	502(26.3)
T3	558(29.2)
T4	148(7.7)
Tx	30(1.6)
N stage	
N0	1700(89.0)
N1-3	210(11.0)
M stage	
M0	1784(93.4)
M1	126(6.6)
Surgery	
Yes	1756(91.9)
No	154(8.1)
Tumor size	
≤ 3 cm	772(40.4)
>3 cm	646(33.8)
Unknown	492(25.8)
Adjuvant therapy	
None	1764(92.4)
Radiation	146(7.6)
Treatment options	
None but conservative treatment	129(6.8)
Surgery only	1635(85.6)
Radiation only	25(1.3)
Both surgery and radiation	121(6.3)
Cause of Death	
Alive or dead from other cause	1206(63.1)
Dead (attributable to transitional cell carcinoma of the urethra)	704(36.9)
Vital status	
Alive	924(48.4)
Dead	986(51.6)

SEER: Surveillance, Epidemiology and End Results.

^a^ Data are presented as the number (percentage) of patients.

### Overall survival and cancer-specific survival

For the entire cohort, there were 986 (51.6%) patients who died and 704 (36.9%) patients who died from primary transitional cell carcinoma of the ureter. The median overall survival (OS) was 46 months, and the 5-year OS rate was 41.8%. The median cancer-specific survival (CSS) was 78 months, and the 5-year CSS rate was 54.3%.

### Univariate and multivariate analysis on overall survival and cancer-specific survival

For demographic, clinicopathological and treatment variables, age, tumor grade, tumor size, AJCC stage, T stage, N stage, M stage, surgery and adjuvant therapy were identified as independent factors for predicting OS in univariate analysis. When multivariate analysis with Cox regression was performed, the variables that were validated as independent prognostic factors included: age range of 65-74 years (hazard ratio (HR): 1.370, 95% CI: 1.124-1.669, *P*=0.002), age range of 75-79 years (HR: 2.022, 95% CI: 1.641-2.493, *P*<0.001), age range of >80 years (HR: 2.695, 95% CI: 2.221-3.272, *P*<0.001), poorly differentiated tumor grade (HR: 1.549, 95% CI: 1.095-2.191, *P*=0.013), undifferentiated tumor grade (HR: 1.807, 95% CI: 1.283-2.546, *P*=0.001), tumor size > 3 cm (HR: 1.174, 95% CI: 1.007-1.369, *P*=0.040), AJCC stage III (HR: 1.790, 95% CI: 1.115-2.874, *P*=0.015), AJCC stage IV (HR: 2.508, 95% CI: 1.557-4.041, *P*<0.001), M stage of M1 (HR: 1.980, 95% CI: 1.513-2.591, *P*<0.001), surgery positivity (HR: 0.411, 95% CI: 0.326-0.517, *P*<0.001), and radiation adjuvant therapy (HR: 1.463, 95% CI: 1.186-1.803, *P*<0.001) (Table 2). The results for predicting CSS were consistent with those for OS, which are shown in Table 3.

**Table 2:** Univariate and multivariate survival analyses of OS in primary transitional cell carcinoma of the ureter from the SEER database

Variable	Univariate analysis		Multivariate analysis	
	HR (95% CI)	*P*	HR (95% CI)	*P*
Age (years)				
<65	Reference		Reference	
65-74	1.367 (1.123-1.663)	0.002	1.370 (1.124-1.669)	0.002
75-79	1.910 (1.554-2.348)	<0.001	2.022 (1.641-2.493)	<0.001
≥80	2.601 (2.150-3.145)	<0.001	2.695 (2.221-3.272)	<0.001
Race				
White	Reference			
Black	1.135 (0.823-1.565)	0.439		
Other (American Indian/AK Native, Asian/Pacific Islander)	0.971 (0.783-1.204)	0.789		
Gender				
Male	Reference			
Female	0.933 (0.823-1.059)	0.283		
Grade				
Well differentiated; Grade I	Reference		Reference	
Moderately differentiated; Grade II	1.084 (0.750-1.566)	0.667	1.122 (0.776-1.624)	0.540
Poorly differentiated; Grade III	2.320 (1.654-3.255)	<0.001	1.549 (1.095-2.191)	0.013
Undifferentiated; anaplastic; Grade IV	2.248 (1.608-3.144)	<0.001	1.807 (1.283-2.546)	0.001
Laterality				
Right - origin of primary	1.045 (0.922-1.184)	0.493		
Left - origin of primary	Reference			
Tumor size				
≤ 3 cm	Reference		Reference	
>3 cm	1.253 (1.078-1.456)	0.003	1.174 (1.007-1.369)	0.040
Unknown	1.483 (1.273-1.728)	<0.001	1.250 (1.063-1.469)	0.007
AJCC stage				
I	Reference		Reference	
II	1.184 (0.980-1.432)	0.080	1.205 (0.656-2.213)	0.549
III	2.377 (1.994-2.832)	<0.001	1.790 (1.6115-2.874)	0.016
IV	4.279 (3.582-5.112)	<0.001	2.508 (1.557-4.041)	<0.001
T stage				
T1	Reference		Reference	
T2	1.135 (0.949-1.359)	0.167	0.968 (0.541-1.730)	0.912
T3	2.336 (1.990-2.743)	<0.001	1.215 (0.781-1.892)	0.388
T4	3.794 (3.043-4.731)	<0.001	1.238 (0.790-1.939)	0.352
Tx	6.001 (4.002-8.999)	<0.001	0.734 (0.429-1.253)	0.257
N stage				
N0	Reference		Reference	
N1-3	2.588 (2.181-3.070)	<0.001	0.912 (0.672-1.237)	0.553
M stage				
M0	Reference		Reference	
M1	4.740 (3.869-5.806)	<0.001	1.980 (1.513-2.591)	<0.001
Surgery				
Yes	0.260 (0.216-0.314)	<0.001	0.411 (0.326-0.517)	<0.001
No	Reference		Reference	
Adjuvant therapy				
None	Reference		Reference	
Beam radiation or radioisotopes	2.213 (1.814-2.699)	<0.001	1.463 (1.186-1.803)	<0.001

OS: overall survival; HR: hazard ratio; CI: confidence interval; SEER: Surveillance, Epidemiology and End Results.

**Table 3:** Univariate and multivariate survival analyses of CSS in primary transitional cell carcinoma of the ureter from the SEER database

Variable	Univariate analysis		Multivariate analysis	
	HR (95% CI)	*P*	HR (95% CI)	*P*
Age (years)				
<65	Reference		Reference	
65-74	1.310 (1.048-1.637)	0.018	1.309 (1.044-1.641)	0.020
75-79	1.715 (1.352-2.177)	<0.001	1.7842 (1.445-2.348)	<0.001
≥80	2.096 (1.680-2.616)	<0.001	2.230 (1.779-2.796)	<0.001
Race				
White	Reference			
Black	0.965 (0.642-1.451)	0.864		
Other (American Indian/AK Native, Asian/Pacific Islander)	1.038 (0.810-1.329)	0.770		
Gender				
Male	Reference			
Female	0.926 (0.797-1.075)	0.310		
Grade				
Well differentiated; Grade I	Reference		Reference	
Moderately differentiated; Grade II	1.365 (0.762-2.447)	0.296	1.3672 (0.764-2.463)	0.289
Poorly differentiated; Grade III	4.452 (2.601-7.620)	<0.001	2.592 (1.502-4.472)	0.001
Undifferentiated; anaplastic; Grade IV	4.399 (2.579-7.506)	<0.001	3.178 (1.84-5.462)	<0.001
Laterality				
Right - origin of primary	1.039 (0.896-1.204)	0.614		
Left - origin of primary	Reference			
Tumor size				
≤ 3 cm	Reference		Reference	
>3 cm	1.492 (1.249-1.783)	<0.001	1.351 (1.125-1.622)	0.001
Unknown	1.637 (1.362-1.969)	<0.001	1.367 (1.125-1.660)	0.002
AJCC stage				
I	Reference		Reference	
II	1.462 (1.138-1.878)	0.003	1.358 (0.693-2.659)	0.372
III	3.461 (2.764-4.334)	<0.001	2.108 (1.253-3.546)	0.005
IV	6.978 (5.582-8.722)	<0.001	3.172 (1.874-5.369)	<0.001
T stage				
T1	Reference		Reference	
T2	1.336 (1.061-1.683)	0.014	1.002 (0.535-1.875)	0.996
T3	3.249 (2.658-3.972)	<0.001	1.353 (0.843-2.171)	0.211
T4	5.614 (4.330-7.279)	<0.001	1.378 (0.852-2.228)	0.191
Tx	10.176 (6.686-15.486)	<0.001	0.907 (0.520-1.582)	0.731
N stage				
N0	Reference		Reference	
N1-3	3.284 (2.730-3.951)	<0.001	0.882 (0.633-1.218)	0.446
M stage				
M0	Reference		Reference	
M1	6.308 (5.100-7.803)	<0.001	2.112 (1.590-2.805)	<0.001
Surgery				
Yes	0.219 (0.179-0.269)	<0.001	0.365 (0.282-0.472)	<0.001
No	Reference		Reference	
Adjuvant therapy				
None	Reference		Reference	
Beam radiation or radioisotopes	2.520 (2.022-3.142)	<0.001	1.521 (1.207-1.918)	<0.001

CSS: cancer specific survival; HR: hazard ratio; CI: confidence interval; SEER: Surveillance, Epidemiology and End Results.

### Effect of treatment options on overall survival and cancer-specific survival

**Figure 1:**
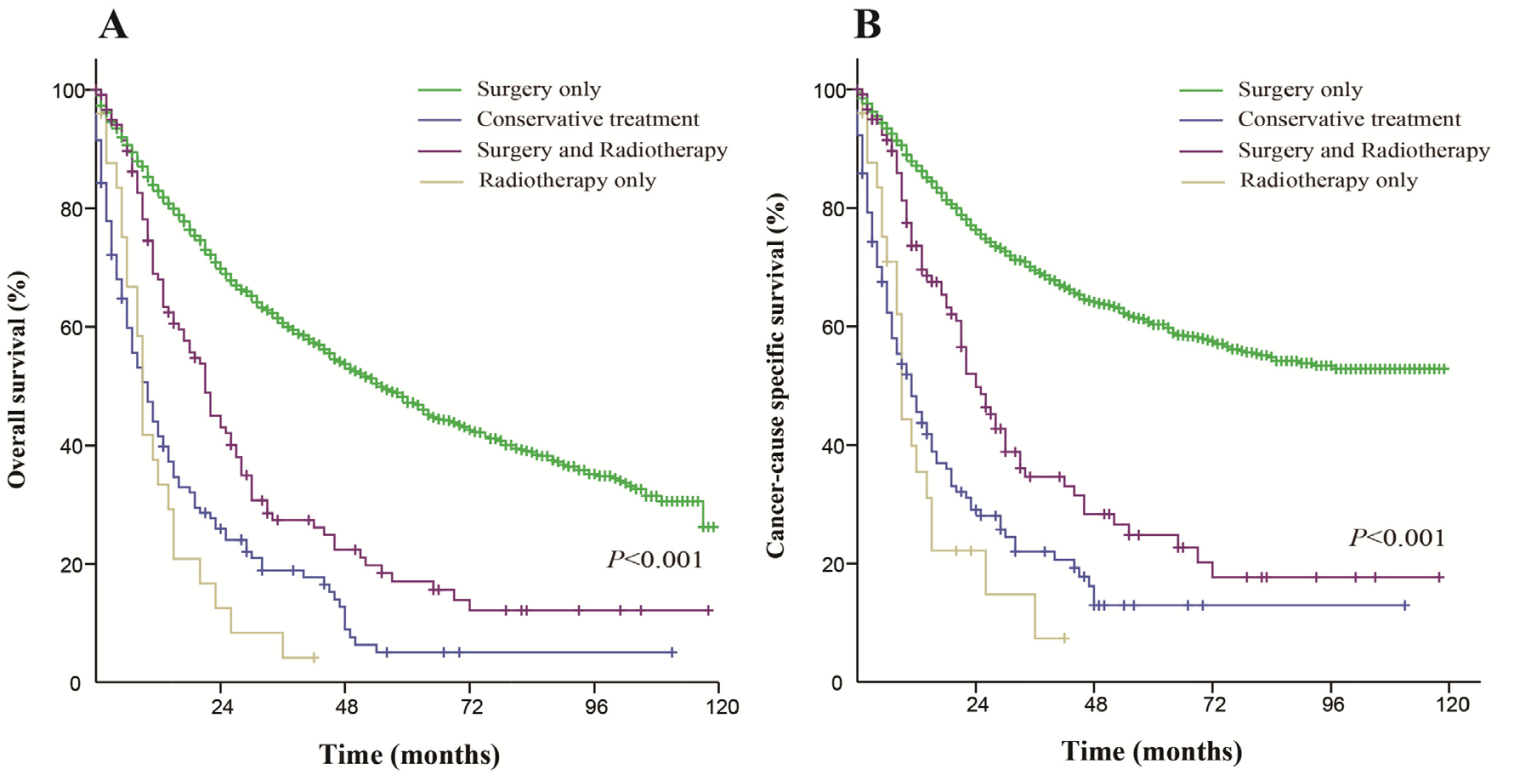
Kaplan-Meier survival curves The overall survival (**A**) and cancer-caused specific survival (**B**) distributions for the treatment options in patients with primary transitional cell carcinoma of the ureter.

Patients who received surgery only had a better OS and CSS than those who received the other therapeutic approaches. The median OS was 55 months in the surgery only group (5-year OS rate was 47.2%), 10 months in the conservative treatment group (5-year OS rate was 5%), 9 months in the radiation only group (5-year OS rate was 0%) and 21 months in the both treatments group (5-year OS rate was 15.6%). The median CSS did not achieve the required 50% survival value in the surgery only group (the 5-year rate was 60.3%), and the median CSS was 11 months in the conservative treatment group (the 5-year CSS rate was 12.9%), 9 months in the radiation only group (the 5-year CSS rate was 0%) and 24 months in the both treatments group (the 5-year CSS rate was 22.7%). These differences were statistically significant according to the univariate log-rank test (*P* < 0.001). The Kaplan-Meier-estimated OS and CSS distributions for the treatment options are shown in Figure 1.

**Figure 2:**
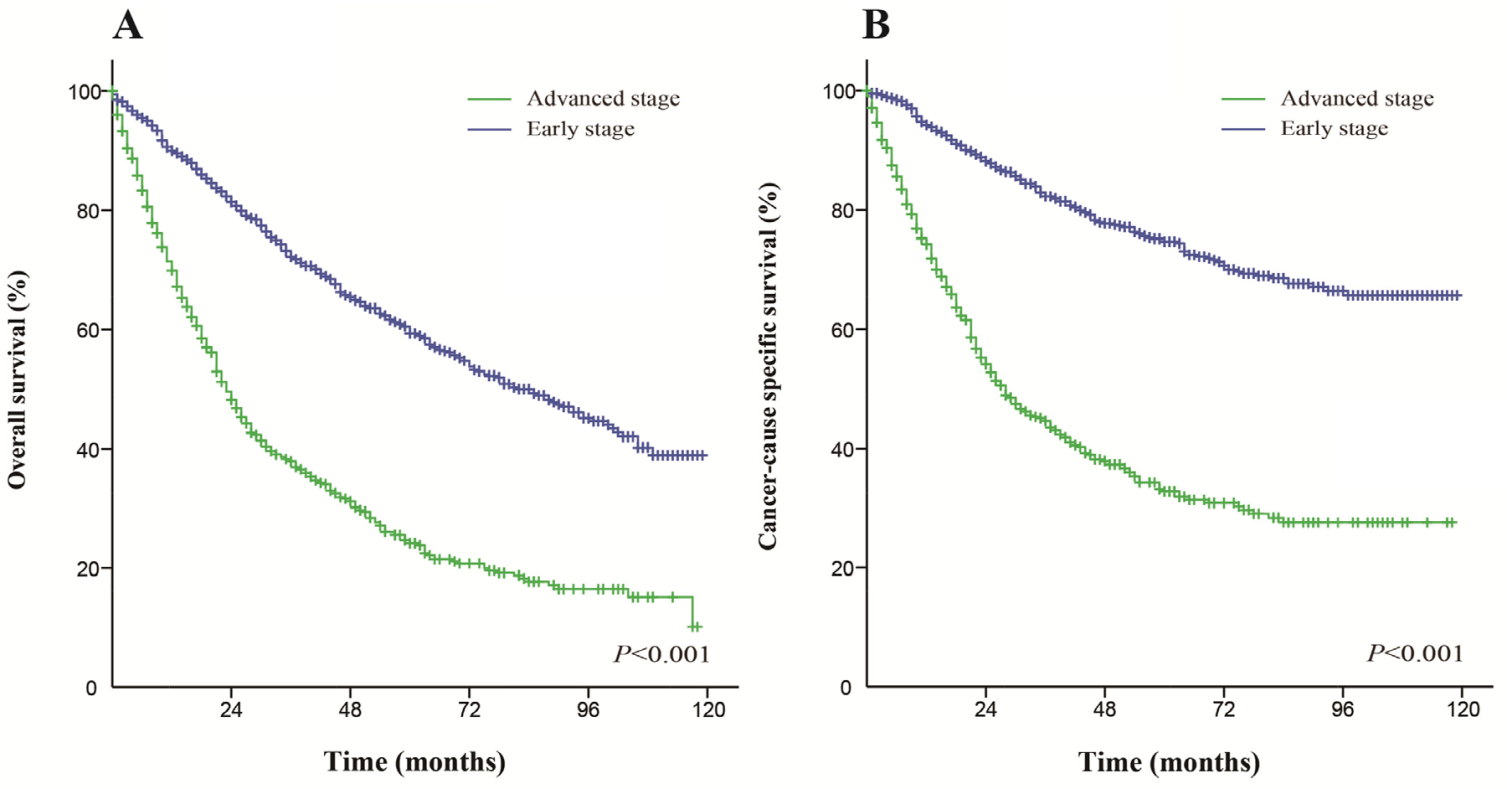
Kaplan-Meier survival curves The overall survival (**A**) and cancer-caused specific survival (**B**) in patients who received surgery.

For patients who received surgery, early stage patients (AJCC stages I-II) had a better OS and CSS than advanced stage patients (AJCC stages III-IV). The median OS was 82 months for early stage patients and 23 months for advanced stage patients. The 5-year OS rate for early stage and advanced stage patients were 59.3% and 24.1%, respectively (Figure 2A). The median CSS was not reached for early stage patients and was 28 months for advanced stage patients. The 5-year CSS rate for early stage and advanced stage patients were 74.7% and 32.8%, respectively (Figure 2B). These differences were statistically significant according to the univariate log-rank test (*P* < 0.001).

**Figure 3:**
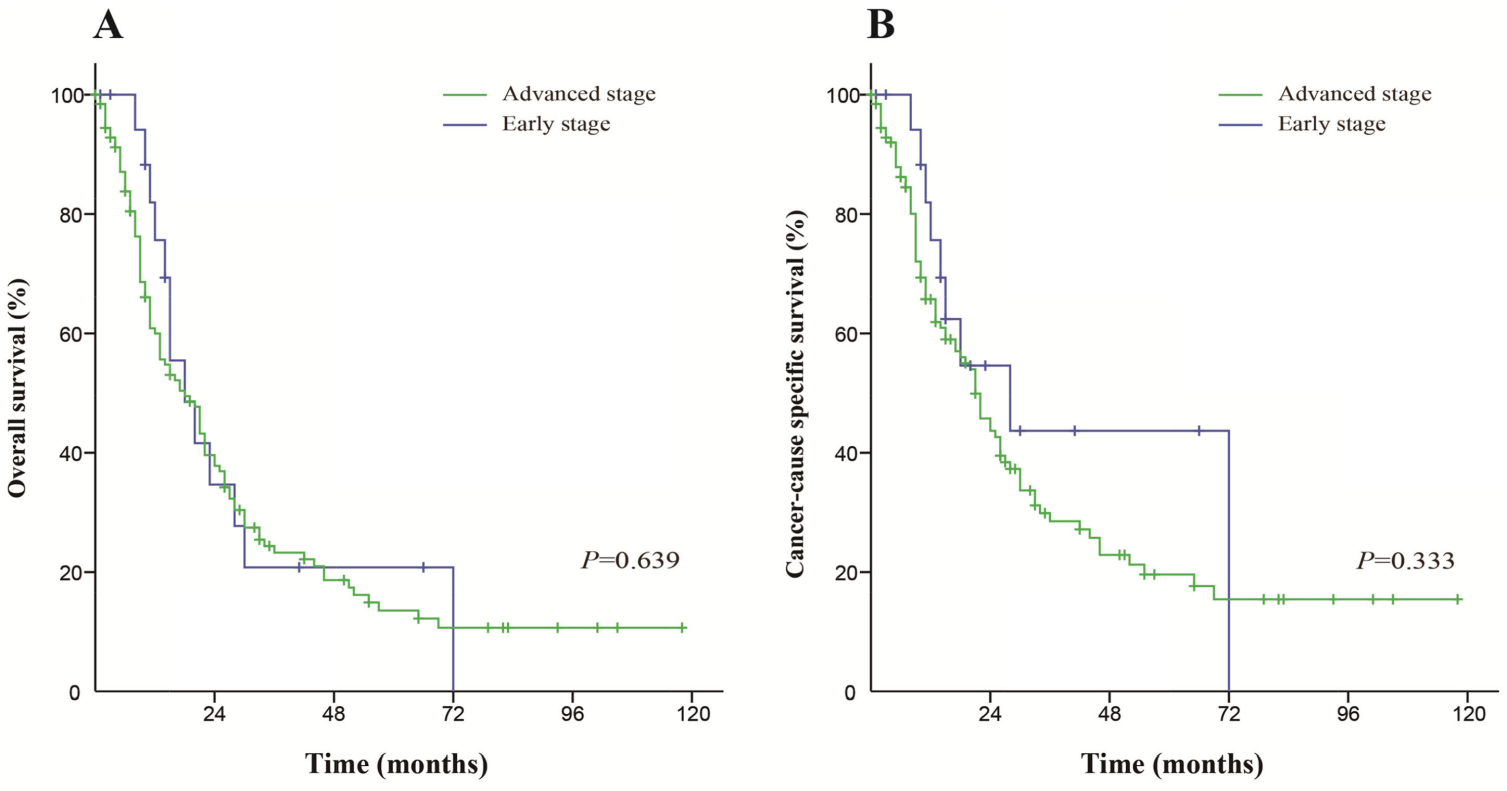
Kaplan-Meier survival curves The overall survival (**A**) and cancer-caused specific survival (**B**) in patients who received radiation.

For patients who received radiation, the differences in the OS and CSS between early stage and advanced stage patients were not significant (*P*=0.639 and *P*=0.330, respectively). The Kaplan-Meier curves of this analysis are shown in Figure 3.

### Effect of demographic, clinicopathological and treatment characteristics for early stage and advanced stage patients

The median OS was 76 months for early stage patients and 21 months for advanced stage patients, while the median CCS was not reached and 24 months, respectively. Tumor differentiations that were defined as poorly differentiated (OR: 9.387, 95% CI: 5.141-17.139, *P*<0.001) and undifferentiated (OR: 6.862, 95% CI: 3.781-12.452, *P*<0.001), with a tumor size of more than 3 cm (OR: 1.300, 95% CI: 1.038-1.672, *P*=0.022), were found to be risk factors for patients with a more advanced stage of cancer using binary logistic analysis (Table 4).

**Table 4:** Logistic analysis of the demographic and clinicopathological factors of the advanced stage of the disease (AJCC stages III and IV) in primary transitional cell carcinoma of the ureter from the SEER database

Variable	OR (95% CI)	*P*
Age (years)		
<65	Reference	
65-74	1.125 (0.863-1.469)	0.384
75-79	1.097 (0.811-1.482)	0.548
≥80	1.029 (0.778-1.360)	0.841
Race		
White	Reference	
Black	1.288 (0.767-2.162)	0.338
Other (American Indian/AK Native, Asian/Pacific Islander)	0.888 (0.648-1.219)	0.464
Gender		
Male	Reference	
Female	1.045 (0.857-1.275)	0.663
Grade		
Well differentiated; Grade I	Reference	
Moderately differentiated; Grade II	1.272 (0.660-2.455)	0.472
Poorly differentiated; Grade III	9.387 (5.141-17.139)	<0.001
Undifferentiated; anaplastic; Grade IV	6.862 (3.781-12.452)	<0.001
Tumor size		
≤ 3 cm	Reference	
>3 cm	1.300 (1.038-1.672)	0.022
Laterality		
Right - origin of primary	Reference	
Left - origin of primary	0.865 (0.713-1.050)	0.142

OR: odds ratio; CI: confidence interval; SEER: Surveillance, Epidemiology and End Results.

**Figure 4:**
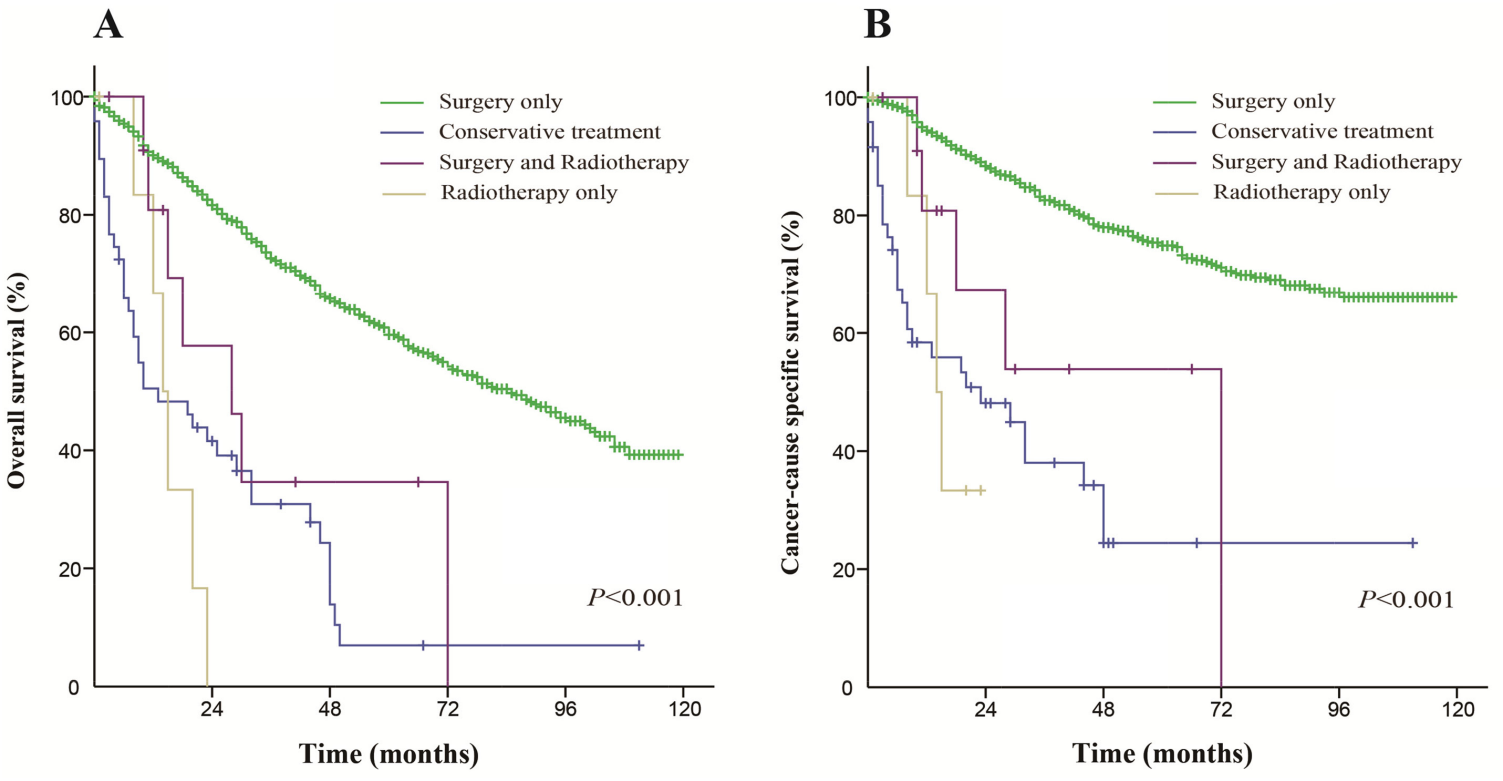
Kaplan-Meier survival curves The overall survival (**A**) and cancer-caused specific survival (**B**) distributions for the diverse treatments in patients with an early stage of the disease.

For early stage patients, the median OS was 85 months in the surgery only group (5-year OS rate was 59.6%), 13 months in the conservative treatment group (5-year OS rate was 7%), 14 months in the radiation only group (5-year OS rate was 0%) and 28 months in the both treatments group (5-year OS rate was 0%). The median CSS was not reached in the surgery only group (the 5-year CSS rate was 74.9%), but was 23 months in the conservative treatment group (the 5-year CSS rate was 24.4%), 14 months in the radiation only group (the 5-year CSS rate was 0%) and 72 months in the both treatments group (the 5-year CSS rate was 0%). These differences were statistically significant (*P* < 0.001), and the Kaplan-Meier curves of the analysis are shown in Figure 4.

**Figure 5:**
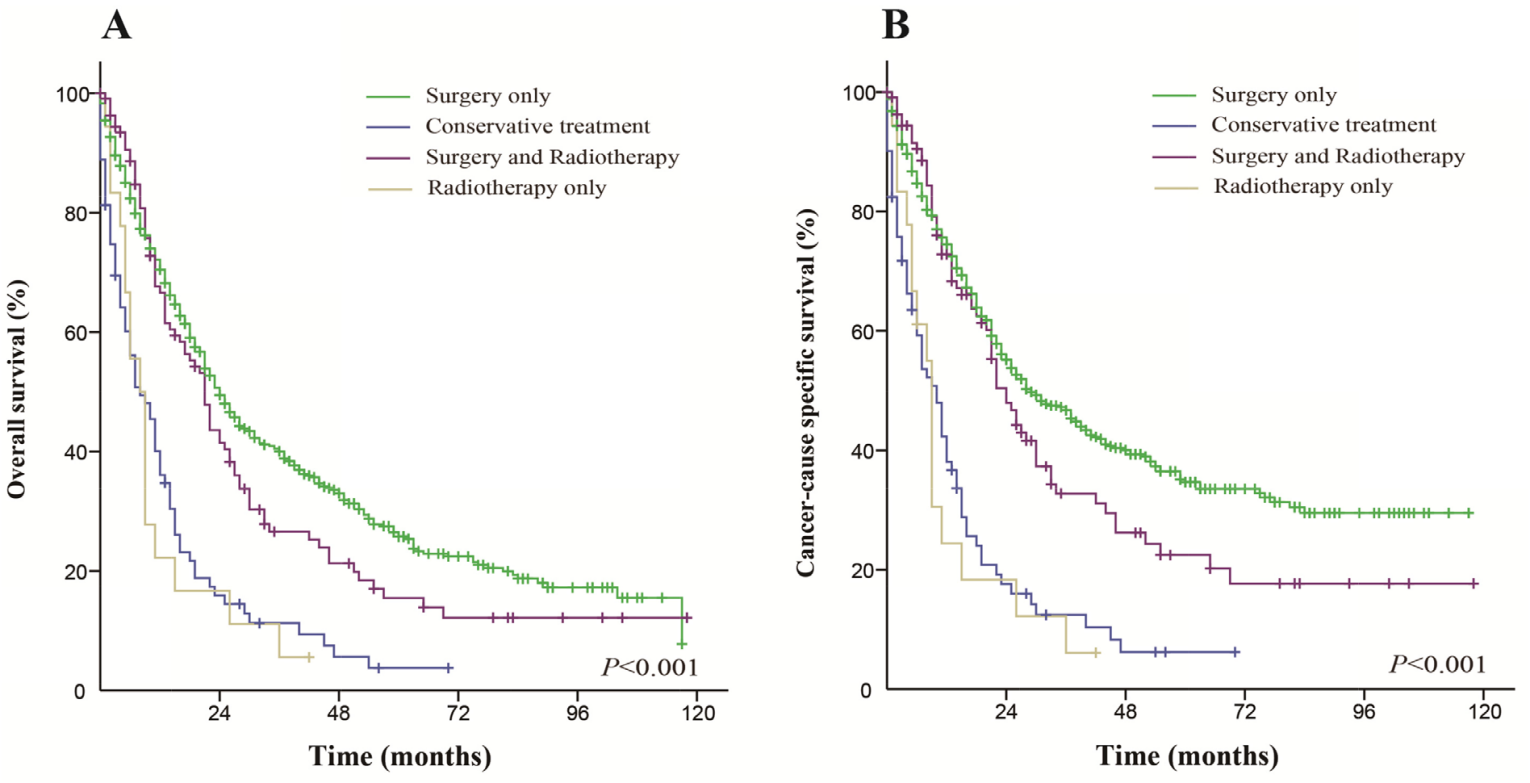
Kaplan-Meier survival curves The overall survival (**A**) and cancer-caused specific survival (**B**) distributions for the diverse treatments in patients with an advanced stage of the disease.

For advanced stage patients, the median OS was 24 months in the surgery only group (5-year OS rate was 25.8%), 8 months in the conservative treatment group (5-year OS rate was 3.8%), 8 months in the radiation only group (5-year OS rate was 0%) and 21 months in the both treatments group (5-year OS rate was 13.9%). The median CSS was 29 months in the surgery only group (the 5-year CSS rate was 34.7%), 10 months in the conservative treatment group (the 5-year CSS rate was 6.2%), 9 months in the radiation only group (the 5-year CSS rate was 0%) and 24 months in the both treatments group (the 5-year CSS rate was 20.2%). These differences were statistically significant (*P* < 0.001), and the Kaplan-Meier curves of this analysis are shown in Figure 5.

## DISCUSSION

As primary transitional cell carcinoma of the ureter is rare, few studies focusing on this disease have been reported. Most studies of UTUCs are limited to either a cohort of a small number of patients or relatively old population-based data. In the present study, we evaluated a large cohort of patients with primary transitional cell carcinoma of the ureter from the new the Surveillance, Epidemiology, and End Results (SEER) database (2004-2013). The median OS was 46 months, and the median CSS was 78 months. The 5-year OS and CSS rates were 41.8% and 54.3%, respectively, as half of the patients were at an early stage of cancer. While additional radiation could not prolong clinical survival, patients with poor tumor differentiation and a large tumor size were more likely to be at an advanced stage of the disease. For early stage patients, the cancer-directed surgical procedure was associated with a fairly longer survival and additional radiation may cause more harm than benefit. Meanwhile, for advance stage patients, the impact of surgery on the OS and CSS declined greatly. Additional radiation had a very small impact on the clinical outcomes of patients. These findings further consolidated the conclusions that were drawn by Margulis [7] and Lughezzani [8], as durable cancer control can be expected in patients with localized UTUC who were treated with a surgical procedure.

The cancer-directed surgical procedure is the most recommended and efficient therapeutic approach at the moment, while physicians still seek to identify a good adjuvant treatment as a supplementary therapeutic approach. As adjuvant treatment modalities have, in fact, been lacking, radiotherapy and chemotherapy have been hot areas of research for several years. Hahn noted that adjuvant radiotherapy may not influence survival among patients with locoregional UTUC, which was shown in a study of 2572 patients regarding the effect of adjuvant radiotherapy on patients with locoregional UTUC [9]. Huang’s observations are consistent with Hahn’s, as adjuvant radiotherapy did not offer any significant benefit in terms of overall, cancer-specific, and recurrence-free survival rates in patients with locally advanced UTUC [10]. This conclusion seems to have been maintained for decades, since 1996 [11–14]. Our findings concerning primary transitional cell carcinoma of the ureter based on a new database have partially updated the former conclusion. Radiation is considered to be an independent prognostic factor in this study, in which it is associated with inferior survival. For the early stage patient cohort, it seemed that radiation caused more harm than benefit on cancer survival, even for patients who already had surgical procedures. Additionally, for patients with an advanced disease, radiation had little impact, which is consistent with the Guidelines on Urothelial Carcinomas of the Upper Urinary Tract (version 2015) [1]. Therefore, it appears unnecessary to perform radiation as one of primary therapeutic approaches for patients with primary transitional cell carcinoma of the ureter, especially for patients with an early stage of the disease. However, this is not a very strong conclusion as there are limitations of the SEER dataset. For example, confounding by indication can obscure any benefit received by radiation because patients with more advanced tumors or additional medical comorbidities might be more likely to receive radiation and have a shorter survival time, for which the limitations and heterogeneity of the data prevent conclusions from being made regarding the effectiveness of the therapeutic approaches. Studies of adjuvant chemotherapy for UTUC have had negative results, such as those on radiation [15–18]. Studies have found no significant difference in the survival of patients, regardless of the administration of adjuvant chemotherapy [19–22]. Traditional chemotherapeutic regimens, such as methotrexate, vinblastine, doxorubicin, cisplatin or gemcitabine and cisplatin, cannot offer as strong of a support as we would like. Cohen indicated that a few patients underwent neoadjuvant chemotherapy, which is defined as surgery within 180 days after the first chemotherapy claim. Neoadjuvant chemotherapy patients showed a better CSS than surgery only patients, while the differences did not reach statistical significance because of the relatively small sample size [23]. Likewise, with few patients undergoing neoadjuvant therapy, there was no demonstrable survival advantage for this approach. We advise caution when drawing conclusions about the effect of neoadjuvant therapy from these limited data. Hence, targeted therapies based on molecular alterations require further investigation.

The principle strength of our study is the latest patient group and large sample size in a population-based setting that is highly representative of practice today in the US. Large population databases can provide answers to questions regarding the incidence, mortality and outcomes of rare malignancies that collaborative or single institutions may not be able to answer. However, there are also a few potential limitations of our study. First, our results could not be extended to patients from Asia, African, Latin America, or Europe or to patients with primary transitional cell carcinoma of the renal pelvis. Second, our study lacked data related to the surgical procedure and radiation details, such as the surgical procedure and radiation type, radiation doses, and treatment techniques, which can vary significantly among institutions [9, 24]. Third, the values reflected by the treatment effect estimates cannot be accurately measured.

In conclusion, our population-based analysis suggests that the cancer-directed surgical procedure is the best choice of treatment for primary transitional cell carcinoma of the ureter and that radiation is not strongly recommended. It also confirms that the current AJCC staging system, as well as the TNM system, is accurate for predicting the prognosis of patients. Beyond this system, an advanced tumor stage is associated with worse clinical survival, and poor tumor differentiation or a large tumor size may lead to an advanced tumor stage. Interpretations of our results are restricted by the inherent limitations of the SEER database. However, this study contributes to the published data by describing the clinicopathological variables, treatments delivered and outcomes for a large cohort of patients, complementing the previously reported studies. Our study demonstrates a requirement for improved adjuvant treatment options for primary transitional cell carcinoma of the ureter and UTUCs. Future studies should focus on this, including multimodality treatment approaches through prospective clinical trials.

## MATERIALS AND METHODS

### Data source

This study used SEER database, which includes data from 18 population-based registries from 1973 to 2013 and covers approximately 30% of the population in the US and was released in November 2015, as its data source. The SEER program registries routinely collect data on patient demographics, primary tumor site, tumor morphology, and tumor stages at diagnosis, first course of treatment, and follow-up for vital status determination. The mortality data reported by SEER are provided and updated annually by the National Center for Health Statistics [25]. The National Cancer Institute’s SEER*Stat software (Surveillance Research Program, National Cancer Institute SEER*Stat software, www.seer.cancer.gov/seerstat) (Version 8.3.4) was used to collect the necessary data.

### Inclusion criteria

To identify appropriate patients for this study, the inclusion criteria for the study were as follows: patients who were diagnosed from 2004 to 2013 with complete clinical manifestations, especially with information from the 6th and 7th versions of the AJCC stage, cancer-directed surgery and radiation; patients who had the ureter listed as the primary disease site (code C66.9); and patients who were diagnosed as having primary transitional cell carcinoma of the ureter (International Classification of Diseases for Oncology, 3rd Edition [ICD-O-3], codes 8120/3: Transitional cell carcinoma, NOS and 8130/3: Papillary transitional cell carcinoma). All patient diagnoses were histologically proven and were regularly followed up. The patients were excluded if they: had an insufficient or unknown clinicopathologic or treatment profile, had an unknown cause of death or unknown survival months, or were diagnosed before 2004. A total of 1910 patients were included in our study.

### Variables for the analyses

The variables that were extracted from the SEER database included age at diagnosis, sex, race, tumor differentiation, tumor laterality, tumor size, AJCC stage, TNM stage, therapy modality, OS and CSS. Age at diagnosis was divided into 4 groups: less than 65 years, 65 to 74 years, 75 to 79 years and 80 years or older. Race was classified into African American, non-Hispanic Caucasus, and others (American Indian/AK Native, Asian/Pacific Islander), as provided by the SEER database. Tumor differentiation was categorized as well differentiated, moderately differentiated, poorly differentiated, or undifferentiated. Tumor laterality was classified as a binary variable, with a right origin of the primary tumor or left origin of the primary tumor, while tumor size was classified as tertiary variable and separated into three groups: ≤3 cm, >3 cm and unknown according to the guidelines [26]. TNM stage and AJCC stage were stratified according to the American Joint Committee on Cancer staging guidelines for renal, pelvis and ureter cancers (6th and 7th edition) [27]. Additionally, we defined AJCC stages I-II as early stage and AJCC stages III-IV as advanced stage. Therapy modality was categorized as: none but conservative treatment, surgery only, radiation only and both.

### Outcome measurement

OS and CSS were the outcomes that we were concerned about. OS was defined as the time span from the date of diagnosis to the date of death and was presented as “vital status,” while CSS was defined as the time span from the date of diagnosis to the date of cancer-specific death and was presented as “SEER cause-specific survival.” Death and death attributed to this cancer were treated as separate events. Patients who died from other causes or were still alive at the time of the last follow-up were treated as censored observations.

### Statistical analysis

To study the association of various covariates with the survival outcome, univariate and multivariate Cox proportional hazard models were built. Binary logistic analysis was performed to determine the risk factors of being at a more advanced stage of disease. The OS and CSS rates were calculated by Kaplan–Meier curves and compared by the log-rank (Mantel-Cox) test. All statistical analyses were performed using the Statistical Package for the Social Sciences (SPSS) software version 22 (SPSS, Inc., Chicago, IL, USA). Differences were considered statistically significant when *P* < 0.05.
